# Emotion regulation group skills training: a pilot study of an add-on treatment for eating disorders in a clinical setting

**DOI:** 10.1186/s40337-020-00289-1

**Published:** 2020-04-03

**Authors:** Kristina Holmqvist Larsson, Anna Lowén, Linda Hellerstedt, Linn Bergcrona, Mimmi Salerud, Maria Zetterqvist

**Affiliations:** 1 Department of Child and Adolescent Psychiatry, Region Östergötland, 581 85 Linköping, Sweden; 2 Department of Biomedical and Clinical Sciences, Linköping university, 581 83 Linköping, Sweden

**Keywords:** Emotion-regulation, Skills, Eating disorders, Treatment

## Abstract

**Background:**

Emotion regulation difficulties appear to play a role in the development and maintenance of several eating disorders. This pilot study aimed at examining whether a short add-on group skills training in emotion regulation for young adults with different eating disorders was feasible in a psychiatric clinical setting. We also investigated if the treatment increased knowledge of emotions, and decreased self-reported difficulties with emotion regulation, alexithymia, symptoms of eating disorder, anxiety and depression, as well as clinical impairment.

**Methods:**

Six skills training groups were piloted with a total of 29 participants (*M =* 21.41 years, *SD* = 1.92). The treatment consisted of five sessions dealing with psychoeducation about emotions and emotion regulation skills training. Paired samples *t*-test was used to compare differences between before-and-after measures.

**Results:**

The primary outcomes measures difficulties in emotion regulation (*p* <  0.001) and alexithymia (*p* <  0.001) showed significant improvement after treatment. The total eating disorder score (*p* = 0.009) was also significantly reduced, as was clinical impairment (*p* <  0.001). Acceptance/valued direction, identifying primary emotions and learning about secondary emotions was rated as especially helpful.

**Conclusions:**

This preliminary pilot study showed that group training targeting emotion regulation skills was feasible and appreciated by participants, as well as being potentially promising as an adjunctive treatment for different eating disorders. Further controlled studies are needed.

**Trial registration:**

The study was retrospectively registered NCT04148014 on October 30th 2019.

## Plain English summary

Emotion regulation difficulties appear to play a role in the development and maintenance of several eating disorders. This pilot study aimed at examining whether a short add-on group skills training in emotion regulation for young adults with different eating disorders was feasible in a psychiatric clinical setting. We also investigated if the treatment increased knowledge of emotions, and decreased self-reported difficulties with emotion regulation, alexithymia, symptoms of eating disorder, anxiety and depression, as well as clinical impairment. The treatment consisted of five sessions dealing with psychoeducation about emotions and emotion regulation skills training. Six skills training groups were piloted with a total of 29 participants. Difficulties in emotion regulation and alexithymia showed significant improvement after treatment. The total eating disorder score was also significantly reduced, as was clinical impairment. Acceptance/valued direction, identifying primary emotions and learning about secondary emotions was rated as especially helpful. In conclusion, this preliminary pilot study showed that group training targeting emotion regulation skills was feasible and appreciated by participants, as well as being potentially promising as an adjunctive treatment for different eating disorders. Further controlled studies are needed.

## Background

As human beings we are constantly faced with the challenge of dealing with negative emotions in our everyday life. Our ability to do this greatly effects our well-being and quality of life [1]. Research has shown a clear association between poor ability to handle emotions and various different psychiatric conditions [2], such as anxiety disorders, depression, substance use disorder and borderline personality disorder [3, 4]. Eating disorders (EDs) are no exception. In fact, emotion regulation difficulties appear to play a role in both the development and the maintenance of several EDs [5–7]. In a longitudinal study of 191 patients with anorexia nervosa (AN), it has for example been shown that high levels of emotion dysregulation on discharge from inpatient treatment predicted an increase and maintenance of AN psychopathology, measured by global score on the Eating Disorder Examination, 12 months following discharge. This relationship was independent of body mass index (BMI) or depressive symptoms. AN severity at discharge did not, however, predict later difficulties with emotion dysregulation [5]. A systematic review by Oldershaw et al. [8] found that individuals diagnosed with AN reported poorer awareness and/or low clarity of emotions and more difficulties with emotion regulation, as well as less access to emotion regulation strategies compared to healthy controls. Studies have further shown that individuals with EDs have more maladaptive strategies for regulating emotions, such as avoidance and suppression, and fewer adaptive strategies, such as acceptance and reappraisal [8, 9].

Emotion regulation can be defined as the process where by an individual shapes which emotions they have, when they have the emotion and how they experience and express the emotion [10]. Gratz and Roemer [11] have contributed a clinically useful conceptualisation where emotion regulation is defined by a set of abilities: awareness and understanding of emotions, acceptance of emotions, ability to control behaviour when experiencing negative emotions and ability to use situationally appropriate emotion regulation strategies flexibly. Based on this definition, difficulties with emotion regulation include avoiding, suppressing or judging emotional experiences and acting impulsively in the presence of negative emotional arousal [11]. In other words, emotion regulation strategies include being attentive to one’s emotional state, identifying and labelling emotions, separating emotions from cognitions, understanding the functions of emotions, allowing and not avoiding the emotional reaction and self-validating, reducing judgement of one’s emotional reaction and being able to prevent impulsive behaviours, such as self-harm, aggressive outbursts, binge eating or substance use, when faced with unwanted emotional experiences [11, 12]. Recent studies have found support for the transdiagnostic character of both emotion regulation [13] and alexithymia [9] (i.e. difficulties identifying and describing emotions, in the pathology of EDs), which supports targeting emotion regulation transdiagnostically across the eating disorder (ED) spectrum.

An ability to regulate emotions is advantageous in the treatment of several psychiatric diagnoses [2]. Treatments that target emotion regulation, such as the emotion regulation group therapy (ERGT), have shown positive results in reducing borderline symptom scores, self-harm and difficulties with emotion regulation in women with borderline personality disorder when given as an adjunct to treatment as usual (TAU), compared to a TAU waitlist group [14]. A study by Racine and Wildes [5] provides support for treatments that target emotion regulation difficulties for AN symptomatology. Several therapeutic models emphasise cognitive and affective components in the treatment of AN [15–18], but fewer studies have examined the effects of including adjunctive emotion regulation skills training as an add-on to the recommended treatments of choice for EDs. One such example is the cognitive remediation and emotion skills training (CREST) [19, 20], where inpatients with AN who received CREST in a group setting + TAU were compared to those only receiving TAU on BMI and neuropsychological performance. There were no significant differences between groups on these measures [19]. In a recent study by our research group, we examined the feasibility and effect of an add-on emotion regulation group skills training delivered to a transdiagnostic child and adolescent psychiatric sample, with promising preliminary results on self-reported alexithymia and emotion regulation difficulties [21]. So-called third-wave therapies, such as Dialectical Behaviour Therapy [6, 12], are promising, showing large effect sizes for treating EDs, but are not superior to traditional CBT [22], which is still the treatment of choice for adults, especially for bulimia nervosa (BN) and binge eating disorders [22]. In the light of this, it seems reasonable to explore the feasibility and effect of including an adjunctive emotion regulation skills training in the treatment of different EDs in a clinical setting. The purpose of psychological treatments is generally considered twofold: to decrease psychiatric symptoms; and to increase level of functioning in areas such as relationships, education or occupation (i.e. to be able to pursue one’s long-term goals and values despite the presence of negative emotion. It is therefore of interest to examine the relationship between increased skills in emotion regulation and less functional impairment.

### The present study

This open trial pilot study aimed at exploring whether a short add-on group intervention of skills training in emotion regulation was feasible in a psychiatric outpatient unit for young adults with EDs. We further examined participants’ experience of participating in the skills training, and whether the intervention increased participants’ knowledge of emotions and decreased self-reported difficulties with emotion regulation, alexithymia, ED symptoms, clinical impairment and symptoms of anxiety and depression. We also examined the relationship between changes in difficulties with emotion regulation and functional impairment.

## Method

### Design

The present pilot study used an uncontrolled open trial design to examine the feasability of a skills training in a group format focusing on emotion regulation, and also the participants’ experience and self-reported symptoms before and after the skills training. Assessment was made at baseline (before treatment) and after treatment (five sessions). All patients were recruited from an eating disorder unit that includes patients up to 25 years at an outpatient child and adolescent psychiatric clinic in Linköping, Sweden, during a period from August 2017 to April 2019. Six skills training groups were administrated during the period from August 2017 to June 2019. No screening for suitability or motivation was done prior to group participation.

### Participants

Participants were all female and primarily Caucasian, aged 18 to 24 years (*M =* 21.41, SD = 1.92). Inclusion was transdiagnostic across the ED spectrum. All participants had been diagnosed with an ED according to DSM-5 criteria [23]. The ED was assessed as the primary diagnosis in all cases. For baseline BMI and diagnoses see Table 1. After an initial referral, a first clinical assessment was performed by clinicians at the ED unit. At a second visit patients were assessed with structural ED measures. All the information gathered was then discussed and evaluated by the ED team (which included a physician, dietitian, physiotherapist, psychologist, counseller and nurse) and a DSM-5 [23] ED diagnosis was agreed upon (if applicable) based on results. Exclusion criteria were ongoing psychosis or mania, drug or alcohol abuse or severe suicidality. Participants were recruited through their ordinary therapist, nurse or dietitian. Participants who were identified as having difficulties with emotion regulation and considered likely to benefit from the skills training were asked to participate. In total, 39 participants were initially recruited, of which 29 completed all sessions and filled in both pre and post assessment. Results are based on these 29 participants. No a priori sample size calculation was conducted and recruitment was ended when the sixth skills training group was completed in June 2019. For participants’ demographics see Table 1.

**Table 1:** Participants’ demographics (*n* = 29)

Variables	Frequency (%)
Females	29 (100)
Age, *m* (*sd*)	21.41 (1.92)
Body Mass index, *m* (*sd*)	23.74 (6.30)
DSM-5 diagnoses	
Anorexia nervosa	4 (13.8)
Bulimia nervosa	9 (31.0)
Atypical anorexia nervosa	9 (31.0)
Atypical bulimia nervosa	3 (10.3)
Unspecified Eating disorder	4 (13.8)
Length of TAU before the skills training	
0–6 months	15 (51.7)
6–12 months	7 (24.1)
1–2 years	1 (3.4)
> 2 years	6 (20.7)
Frequency of TAU sessions during the skills training	
0	2 (6.9)
1–5	23 (79.3)
> 5	4 (13.8)

*Note. DSM-5* Diagnostical and Statistical Manual of Mental Disorders, fifth version [24], *TAU* Treatment as Usual

### Ethical considerations

The study was approved by the Regional Ethical Review Board of Linköping (Dnr, 2015/264–31 & 2017/472–32). Participants received oral and written information about the study from their therapist, nurse or dietitian, and also from the skill trainers during the introduction of the first session. All participants signed an informed consent form. Participants were informed that they could withdraw from the skills training at any given moment without any consequences for their ongoing treatment.

### Measures

#### Primary outcome measures

##### Difficulties in emotion regulation scale (DERS)

DERS [11] is a widely used self-reported questionnaire that measures ability to modulate emotional arousal, awareness, understanding and acceptance of emotions, and the ability to engage in a goal-directed behaviour regardless of emotional state. The questionnaire consists of 36 items rated on a five-point Likert scale from “almost never” to “almost always”. Higher scores indicate more difficulties in regulating emotions. DERS consists of a total scale for emotion regulation difficulties, as well as six subscales: Nonacceptance, Goals, Impulse, Awareness, Strategies and Clarity. In the present t-test analysis of within-group differences, the total DERS scale was used. For correlation analysis of pre-post change in difficulties with emotion regulation and functional impairment, the total DERS and all the DERS subscales were used. Cronbach’s alpha for the total scale for the present sample was α = .87, indicating good internal consistency, and for subscale nonacceptance: α = .88, goals: α = .70, impulse: α = .76, awareness: α = .73, strategies: α = .89 and clarity: α = .78. Internal consistency for the subscales ranged from acceptable to good.

##### Toronto alexithymia scale (TAS-20)

TAS-20 [24] measures alexithymia and consists of 20 items, ranging from “totally right” to “totally wrong” on a five-grade Likert scale. The questionnaire comprises three subscales: difficulties identifying emotions; difficulties describing emotions and difficulties externalising emotions. A higher score indicates higher levels of alexithymia. In this study, the total scale was used. TAS-20 is one of the most used self-report scales for alexithymia [25], and has shown good reliability and validity [26, 27]. Internal consistency for the total scale for the present sample was α = .90, indicating excellent internal consistency.

##### Eating disorder examination questionnaire (EDE-Q)

EDE-Q [28, 29] is the self-report version of Eating Disorder Examination (EDE) [30] and measures the characteristics of EDs. The 6.0 version used in this study consists of 28 items. EDE-Q is scored on a 7-point Likert scale, from “no days” to “every day” and higher scores indicate higher eating pathology. The questionnaire comprises a total scale and subscales for restraint, eating concerns, shape concerns and weight concerns. In the current study, the total EDE-Q score and the four subscales were included in the analysis. EDE-Q has shown good psychometric properties [31], and there is support for the reliability and validity of scores on EDE-Q for assessing ED symptoms [32]. Cronbach’s alpha for the present sample was α = .93, indicating excellent internal consistency for the total scale, and corresponding alpha for the subscales was α = .83 (restraint), α = .64 (eating), α = .92 (shape), α = .89 (weight), indicating good to excellent internal consistency for all subscales, except for eating concern, which had questionable internal consistency.

##### The clinical impairment assessment questionnaire (CIA)

CIA [29, 33] measures the psychosocial impairment of EDs. It focuses on the last 28 days and consists of 16 items, graded on a Likert scale from “Not at all” to “A lot”. Higher scores indicate a higher level of secondary psychosocial impairment. CIA is designed to be completed immediately after filling in a measure of current ED features that covers the same time frame (e.g., the EDE-Q). Internal consistency for the present sample was α = .89, indicating good internal consistency for the total scale.

#### Secondary outcome measures

##### Beck anxiety inventory (BAI)

BAI [34] is a commonly used measure of symptoms of anxiety with 21 items rated on a four-point scale. High scores indicate higher levels of anxiety. BAI has good psychometric properties and discriminates anxiety disorders from other diagnoses [34]. Cronbach’s alpha for BAI in this study was excellent, with α = .92.

##### Montgomery Åsberg depression rating scale, self-report version (MADRS-S)

MADRS-S [35] measures symptoms of depression. Its main purpose is to monitor the development of symptoms during treatment. The scale consists of nine items, which are graded from zero to six. Higher scores indicate a higher level of depression symptoms. MADRS-S correlates well (*r* = .87) with Beck Depression Inventory [36]. Internal consistency for MADRS-S in the present sample was α = .90, indicating excellent internal consistency.

### Intervention

The emotion regulation skills training consisted of five two-hour weekly sessions in a group setting. Group sizes varied from three to seven participants. Six skills training groups were administered. The intervention focused on psychoeducation about emotions, the functions of emotions, acceptance of emotions and teaching skills for identifying, labelling, expressing and regulating emotions (Table 2). The intervention was based on treatment principals from Emotion Regulation Group Therapy (ERGT) [37], Unified Protocol (UP) [38], Dialectical Behaviour Therapy (DBT) [12] and Acceptance and Commitment Therapy (ACT) [39] and was developed by Holmqvist Larsson and Zetterqvist [21]. Between each session the participants were given a homework assignment. Each session included a rehearsal of the previous session’s content and a run-through of the homework assignments. The content of each session was introduced by psychoeducation and illustrated with role play/video vignettes, and was followed by a discussion led by the skills trainers. The content was presented using PowerPoint slides and participants also received handouts.

**Table 2:** Description of the emotion regulation skills training

Session	Content	Homework assignment
1	Rationale about emotion regulation What are emotions? Identifying and labelling emotions	Identify and label emotions
2	Emotions and behaviour The functions of emotions Self-validation	Identify emotions, their behavioural impulse and function Self-validation
3	Primary and secondary emotions Expressing primary emotion Validation of others	Expressing primary emotion
4	Reducing vulnerability A mindful choice of opposite action or acting on the emotion	Increasing positive activities Mindful choice of following the impulse or opposite action
5	Staying with the emotion Acceptance and willingness Values	

The four skills trainers in this pilot study (the second, third, fourth and fifth authors) all had a Master’s degree (three clinical psychologists and one CBT psychotherapist) with experience of clinical psychiatry and the treatment of EDs. The trainers received initial training in the method and the structured manual by the first and last authors. Each group was led by two skills trainers.

Nearly all of the participants (*n* = 28, 96.6%) had ongoing TAU during the skills training. Of these, 57.1% had visits to a dietitian, nurse or physician; 14.3% received Fairburn’s CBT-E; 10.7% received support by a counsellor; 7.1% were in interpersonal therapy and 10.7% were seeing a psychologist for traditional CBT. For information about TAU see Table 1. There were no predefined rules to determine at what stage during TAU a participant could participate in the group skills training.

### Consumer satisfaction and impact on knowledge

Written statements which assessed participants’ level of satisfaction and perception of increased knowledge following the skills training were created for the study and filled in by the participants at the last session. They were filled in anonymously and rated on a 5-point Likert scale ranging from agreeing “not at all” to “very much so”. Participants also rated anonymously which of eight content domains (primary emotions; the functions of emotions; secondary emotions; differentiating between emotions thoughts and actions; validation; reducing vulnerability; emotion regulation; acceptance/valued directions) they thought was most helpful and which was most difficult during the skills training. At the end of the last session participants were also encouraged to leave anonymous comments in writing under the headings, describing what they had appreciated and offering suggestions for improvement regarding both the skills training and the skills trainers, and they were also free to leave other comments. The comments were analysed and presented in different categories that described the content. The frequency and percentage of participants who spontaneously left a comment under each category was presented, together with a description of the category and an example.

### Statistical analysis

Descriptive statistics were derived using mean values and standard deviations. Paired samples *t*-test was used to compare before-and-after differences on all self-report measures (DERS, TAS-20, EDE-Q, CIA, MADRS-S and BAI) for the whole group. After conducting multiple t-tests, multiple comparisons were corrected for by dividing the number of comparisons with alpha level 0.05 to receive a more stringent alpha level. Within-group effect size (ES) was calculated using Cohen’s *d* with 0.2, 0.5 and 0.8 indicating small, medium and large ES, respectively. For correlation between changes in difficulties with emotion regulation (DERS) and functional impairment (CIA), Pearson’s product moment correlation coefficient (*r*) was used. Cronbach’s alpha was used for internal consistency. All statistical analyses were performed using the SPSS 24.0 software package.

## Results

### Feasibility

A majority of the participants who came to the first session completed the entire treatment and filled in post-treatment measures (29 of 39, 74.4%). Reasons for dropping out were: disliking the group format (*n* = 2); severe mental health problems that prevented them from completing the skills training (*n* = 1); starting another treatment that was not compatible with the skills training (*n* = 1); moving (*n* = 1); wrong timing (*n* = 1) and reason unknown (*n* = 2). For an additional two individuals, the post-treatment measures were not collected. Those who did not complete the treatment and/or did not fill in post measures (*n* = 10) did not differ from those who followed through (*n* = 29) on total scores of baseline measures of alexithymia, ED symptoms, level of functional impairment, or symptoms of anxiety and depression. The individuals who dropped out did, however, have significantly higher baseline scores of emotion regulation difficulties, *t*(35) = 2.17, *p* = 0.04. After correcting for the multiple comparisons (6/0.05 = 0.008), the difference was no longer statistically significant. There were more individuals with AN (including atypical AN, which a majority had) among those who did not complete treatment (*n* = 8, 80.0%) compared to those who did (*n* = 13, 44.8%), but the difference was not statistically significant. There was no statistical difference in BMI between the groups.

### Outcomes

#### Primary outcome measures

Participants’ self-reported difficulties with emotion regulation, measured with total DERS scores, decreased significantly (*p* <  0.001) following the skills training with a large ES. Participants’ ratings of alexithymia (total TAS-20 scores) were also significantly reduced (*p* <  0.001) with a medium ES (Table 3), which suggests an improved awareness of emotions (i.e. an ability to identify and describe both one’s own emotions and the emotions of others after the emotion regulation skills training). The mean total score of EDE-Q, measuring ED symptoms, was significantly improved (*p* = 0.009) after the skills training with a small ES. Results on the subscale “Restraint”, measuring restraints in eating, did not change however, but the other subscales measuring concerns with eating (*p* = 0.001), shape (*p* = 0.01), and weight (*p* = 0.03) were significantly improved, although with small ES, for all but the subscale “concerns with eating” that showed a medium ES (Table 3). Self-reported clinical impairment was significantly reduced, with a medium ES, following the skills training (*p* <  0.001). See Table 3.

**Table 3:** Participants’ (n = 27–29) self-reported difficulties with emotion regulation, alexithymia, symptoms of depression, anxiety, eating disorder and clinical impairment before and after treatment, means, standard deviations and effect sizes

Measures	Before treatment	After treatment	Stat		
	M (SD)	M (SD)	*t*	*p*	ES
Primary outcomes					
DERS^a^ total	112.19 (16.38)	93.56 (16.42)	5.06	< 0.001	1.14
TAS^a^ total	57.66 (13.47)	48.79 (10.19)	5.68	< 0.001	0.75
EDE-Q total^a^	3.34 (1.20)	2.90 (1.46)	2.80	0.009	0.33
Restraint	2.06 (1.50)	2.06 (1.68)		*ns*	
Eating concern	2.90 (1.15)	2.21 (1.36)	3.78	0.001	0.55
Shape concern	4.56 (1.40)	3.99 (1.76)	2.62	0.01	0.36
Weight concern	3.83 (1.69)	3.33 (1.75)	2.29	0.03	0.29
CIA^a^	27.41 (9.21)	21.79 (10.17)	4.13	< 0.001	0.58
Secondary outcomes					
MADRS-S^a^	21.28 (10.07)	17.59 (9.33)	2.64	0.01	0.38
BAI^a^	19.30 (11.28)	15.89 (10.44)		*ns*	

Note. *DERS* Difficulties with emotion regulation skills, *TAS* Toronto Alexithymia Scale, *EDE-Q* Eating Disorder Examination Questionnaire, *CIA* Clinical Impairment Assessment Questionnaire, *MADRS-S* Montgomery Åsberg Depression Rating Scale -Self-report version, *BAI* Beck Anxiety Inventory, ^a^higher scores indicate more difficulties. Cohen’s *d* effect size (ES) was calculated

#### Secondary outcome measures

Furthermore, there was a significant decrease in symptoms of depression (*p* = 0.01) with a small ES, but symptoms of anxiety, measured with BAI, were not significantly reduced. See Table 3.

After correcting for the multiple comparisons (10/0.05 = 0.005), the only improvement that remained significant was on DERS, TAS-20, EDE-Q subscale eating concern and CIA.

### Difficulties with emotion regulation and functional impairment

There was a significant correlation (*p* = 0.001) between pre-post change on DERS and CIA, indicating that reduced self-reported difficulties with emotion regulation were related to reduced self-reported clinical impairment. This was especially true for the relationship between DERS subscales “goals” (*p* = 0.008), “impulse” (*p* = 0.001), “strategies” (*p* = 0.003), “clarity” (*p* = 0.04) and CIA. See Table 4.

**Table 4:** Correlations between pre-post change in CIA and DERS total and DERS subscales, Pearson correlation

		DERS total	DERS nonacceptance	DERS goals	DERS impulse	DERS awareness	DERS strategy	DERS clarity
CIA	Pearson Correlation	0.620	0.266	0.500	0.624	0.158	0.556	0.401
	Sig. (2-tailed)	0.001	*ns*	0.008	0.001	*ns*	0.003	0.038
	*N*	27	27	27	27	27	27	27

Note. *DERS* Difficulties with emotion regulation skills, *CIA* The Clinical Impairment Assessment Questionnaire

### Increased knowledge

The participants reported that they were very satisfied with the skills training and its content (*M* = 4.78, *SD* = 0.42, on a 5-point scale). They reported that their understanding of their emotions had increased (*M* = 4.29, *SD* = 0.66) and that they had benefited from the skills (*M* = 4.18, *SD* = 0.55). To what extent they had managed to generalise the skills (*M* = 3.57, *SD* = 0.69) and whether the skills training contributed to their recovery from their ED (*M* = 3.32, *SD* = 0.82) received average ratings when participants evaluated their experience of the skills training. See Table 5.

**Table 5:** Participants’ evaluation after the skills training, means and standard deviations, and frequencies and percentages

Items^a^	Participants *n* = 28	
Are you satisfied with the skills training and its content?	4.78 (.42)	
To what extent have you generalised the skills you learnt in the skills training?	3.57 (.69)	
How much benefit have you had of the skills?	4.18 (.55)	
Has the skills training increased your understanding of your own emotions?	4.29 (.66)	
To what extent has the skills training contributed to your recovery from your eating disorder?	3.32 (.82)	
Content domain’	Most helpful *n* (%)	Most difficult *n* (%)
Primary emotions	17 (60.7)	0 (0)
The functions of emotions	12 (42.9)	0 (0)
Secondary emotions	18 (64.3)	4 (14.3)
Differentiating between emotions, thoughts and actions	16 (57.1)	0 (0)
Validation	9 (32.1)	5 (17.9)
Reducing vulnerability	7 (25.0)	0 (0)
Emotion regulation	9 (32.1)	1 (3.6)
Acceptance and valued direction	19 (67.9)	6 (21.4)

*Note.*^a^Scale ranging from 1 “not at all” to 5 “very much so”. ‘Each participant could rate several content domains as helpful and/or difficult

### Participants’ experience of targeting emotion regulation skills in treatment

The top three content domains that participants rated as most helpful were acceptance/valued direction (19 of 28, 67.9%), secondary emotions (18 of 28, 64.3%) and primary emotions (17 of 28, 60.7%). See Table 5. The content was not perceived as very difficult overall, but the domains that received most ratings as being difficult were acceptance/valued direction (6 of 28, 21.4%), validation (5 of 28, 17.9%) and secondary emotions (4 of 28, 14.3%). Participants’ written anonymous comments were categorised under the following six headings: Emotion regulation skills content; Group format; Structure; Pedagogical aspects; TAU; Skills trainers. See Table 6 for description and examples.

**Table 6:** Participants’ experiences of the emotion regulation skills training, *n* = 24

Category		Description	*n* (%)	Example
Emotion regulation skills content	+	Interesting, important and helpful	14 (58.3)	“Good group with good aim which has made it easier for me to cope with my emotions, and understand and verbalise what I am feeling (most of the time)”
	–	Difficult	2 (8.3)	“Difficult but fun to work with this at home”
Group format	+	Sharing and listening to each others’ experiences in a safe environment.	10 (41.7)	“There was a safe atmosphere in the group and one dared to share one’s experiences. Beforehand, I was scared of meeting the others and having to share experiences with them, but now I almost wish that there had been more opportunities to discuss with the others and exchange thoughts. It’s interesting to listen to others in the same situation as oneself and very comforting to feel that one is not alone with this…”
	–	Preferring individual format	2 (8.3)	“I think it’s very difficult in a new group to talk about this when everyone has different backgrounds and difficulties. I think it would have given me more to do it individually in order for it to have the most effect. So that one in a different way could relate it to one’s everyday life”
Structure	+	Right amount of time and length	3 (12.5)	“Right amount of information in each session to be able to work on it. Number of sessions, pace and content has been good”
	–	More sessions and time to practice	9 (37.5)	“…alternatively one could have had more sessions so that the information could have sunk in more and there would have been more time to practise on one’s own”
	–	Too many and too long sessions	3 (12.5)	“Since the sessions are quite long, it sometimes felt a bit slow and one got a bit tired, difficult to concentrate”
Pedagogical aspects	+	Mixture of lectures, PowerPoint, exercises, discussion, film clips	8 (33.3)	“Good setup, partly with PowerPoint where you gave us an insight into what we were going to talk about and one gained knowledge. So as to tackle the subject oneself afterwards and to evaluate it in exercises, for example.”
	+	Homework	4 (16.7)	“The discussions and homework were good because then one took in everything one had learnt in a better way into everyday life.”
	–	More in depth discussion	4 (16.7)	“Could have been a bit more and a bit deeper knowledge”
Treatment as usual (TAU)	+	Incorporating the skills training in TAU	1 (4.2)	“Now it’s possible to complement the sessions with one’s regular treatment and therapy instead, which also works fine!”
	–	Wrong timing of skills training	1 (4.2)	“It has been a lot to take in during five sessions, and as relatively newly diagnosed it has been a lot to digest”
Skills trainers	+	Validating, knowledgeable and active	16 (66.7)	“I like that they have been active and also done the homework, it shows that it is doable and that everyone can do it. It has been easy to ask questions and discuss things with them”
	–	Lacking in knowledge	1 (4.2)	“Sometimes there were some questions that you didn’t really know how to answer, at least that’s what it felt like. Then it got a bit confusing. But most of the time you could answer and explain very well so that one understood”

*Note.* Participants were specifically asked to write comments on the skills trainers. The other categories were created based on participants’ spontaneous written comments on what they appreciated (+) and suggested for improvement (−) concerning the skills training

## Discussion

This pilot study targeted potential underlying difficulties with emotion regulation in EDs by including an adjunctive emotion regulation skills training in a group format in the treatment of a clinical sample of young adults with different EDs. This short add-on intervention was feasible in an ED unit and appreciated by participants. Results showed potential promise with significantly reduced self-reported difficulties with emotion regulation, alexithymia, ED scores and general clinical impairment following the skills training.

Participants’ levels of self-reported alexithymia and difficulties with emotion regulation before the skills training in this pilot study confirm earlier studies, which show high rates of alexithymia measured with TAS in ED populations [13], as well as difficulties with emotion regulation measured with DERS [40, 41]. The mean average of total DERS scores in an earlier study by Monell and colleagues [40] was 102.1 (SD = 26.3) in a Swedish psychiatric ED sample, compared to a mean average of 112.19 (SD = 16.38) in our sample. The high level of difficulties with emotion regulation in our ED sample is most probably explained by the fact that participants had already been identified as having difficulties with emotion regulation and were therefore considered to be in need of the skills training and likely to benefit from it. This also highlights the potential importance of targeting emotion regulation in the treatment of EDs. An earlier study based on the Stepwise register in Sweden on clinical females 18–66 years (*N* = 2, 383) with diagnosed ED [29] had an EDE-Q average global score of 4.06 (*SD* = 1.20) compared to *M* = 3.34 (*SD* = 1.20) in our study. Results on EDE-Q in the study by Welch and colleagues [29] varied between different EDs, with BN showing significantly higher total scores compared to other EDs, such as ED not otherwise specified (NOS) and AN which showed the lowest scores. In our sample, a majority (62.1%) of participants had AN, atypical AN or ED NOS, which is a possible explanation for the somewhat lower EDE-Q scores in our sample. Subgroup analyses based on ED diagnosis in this study was not possible, however, due to the small sample size.

Interestingly enough, our study found support for improvement, not only regarding symptoms but also clinical impairment, which is an important treatment target [29]. Total scores on CIA decreased significantly from *M* = 27.41 (*SD* = 9.21) before treatment to *M* = 21.79 (*SD* = 10.17), with a medium ES. Comparable data from the Stepwise register on adult EDs in Sweden [29] had a CIA average total score of 30.22 (SD = 10.21). There was also a significant correlation between pre-post change in difficulties with emotion regulation and functional impairment, which potentially indicates that improved emotion regulation is associated with less functional impairment.

Compared to other add-on emotion regulation group treatments, the results from the CREST add-on treatment given to adults with AN [19] are not easily comparable to our results, since Davies and colleagues used neuropsychological performance tests as outcome measures. Participants’ experience of CREST and targeting emotion regulation skills as an add-on intervention in treatment was, however, very similar to that of our participants who appreciated learning about emotions [20]. In our previous transdiagnostic add-on group emotion regulation treatment for adolescents [21], we found medium ES decrease in emotion regulation difficulties and alexithymia compared to large ES for DERS and medium ES for TAS in the present study. Other add-on emotion regulation skills studies targeting emotion regulation skills, such as the ERGT for women with borderline personality disorder, have shown decreases in emotion regulation difficulties with significant effects on DERS with medium ES [14]. Emotion Acceptance Behavior Therapy (EABT) [17], a longer intervention, not given as an add-on, found a large ES decrease in EDE-Q scores before and after treatment. They also found a significant difference in anxiety as measured with BAI, which was not found in the present study. Taken together, data suggests that there seems to be a potential advantage in addressing difficulties with emotion regulation in this patient group.

Clinically, there are benefits of including skills for emotion regulation in the treatment for this population, since more maladaptive strategies for regulating emotions, such as controlling, avoiding and suppressing emotions, are not uncommon in patients with ED [8] and can reinforce the disorder [5]. The participants in this study were positive to targeting emotion regulation as an add-on treatment. They perceived the skills training to be helpful and that they benefited from it. Discriminating between emotions and learning about primary and secondary emotions were rated as especially helpful. In working with secondary emotions, the focus is on reducing judgment and focusing on validating one’s emotional experience. This could possibly represent a new, albeit difficult, and helpful way of viewing oneself instead of turning to self-criticism, which has been shown to be associated with ED symptoms [42]. Participants also appreciated working with acceptance and valued direction, although this was at the same time rated as the most difficult content by some of the participants. Validation was also perceived as somewhat more difficult for some participants. However, some found the group format itself helpful, and listening to others share similar lines of thoughts and emotions seemed to facilitate validation of experiences. Skills training in emotion regulation could be especially meaningful for those where difficulties with emotion regulation have been assessed as maintaining the ED. Increased adaptive skills in emotion regulation, such as becoming aware of emotions, identifying, labelling, expressing and accepting emotions, and furthermore preventing impulsive behaviors, could potentially be one of several facilitators towards recovering from an ED. Although not statistically significant, those who dropped out from the treatment reported higher baseline levels of difficulties with emotion regulation than those who completed the skills training. These participants were therefore in all likelihood in particular need of the skills training. One hypothesis is that the group format was perceived as too demanding, which some participants gave as a reason not to follow through.

The study’s strengths include addressing emotion regulation transdiagnostically within the ED spectrum and conducting a treatment intervention in a naturalistic psychiatric setting with a clinical sample. The pilot study has some limitations that need to be addressed. The lack of a controlled design is a major limitation, making it difficult to draw any conclusions as to whether the improvements noted should be attributed to the skills training or to other ongoing clinical interventions. Thus, many factors could have influenced the outcome, and caution needs to be taken when interpreting the results, as there was no active control group or randomisation. The TAU that continued during this add-on intervention could reasonably also have influenced the results. Future studies are therefore needed which include randomisation and a controlled design. The preliminary positive effects could also be possible therapist effects. It is also likely that the results could be influenced by demand characteristic, as the purpose of the study couldn’t be disguised from the participants. Another limitation is that there was no ongoing supervision during the skills training, which potentially could have reduced adherence to the method. We have no information on how many participants were asked to participate and declined. There could potentially be some participant bias in that those individuals who were interested in targeting emotions agreed to participate in the treatment study (i.e. the sample consisted of more motivated individuals). Those not in favour of working with emotions or not appreciating the group format probably didn’t sign up or dropped out, which could have led to more positive results. Although total results on EDE-Q and CIA were significantly improved following the skills training, scores were still in the clinical range and well above general population scores on these measures [29]. Since the study examined a short pilot intervention in a clinical sample with DMS-5 [23] EDs, delivered during a time frame of 5 weeks, it was not realistic to measure recovery rates at post-treatment. A longer follow-up is also missing.

## Conclusions

This short add-on emotion regulation group skills training intervention was feasible in a psychiatric eating disorder unit and appreciated by participants with different eating disorders. Results showed potential promise with significantly reduced self-reported difficulties with emotion regulation, alexithymia, eating disorder scores and general clinical impairment following the skills training. An adjunctive emotion skills training could proposedly be delivered sequentially after first choice evidence-based recommended interventions have been delivered to stabilise the eating behavior [22].

## Data Availability

The datasets generated and/or analysed during the current study are not available since individual privacy could be compromised.

## Abbreviations

AN: Anorexia nervosa
BAI: Beck anxiety inventory
BN: Bulimia nervosa
CBT: Cognitive behavioural therapy
CIA: Clinical Impairment Assessment Questionnaire
CREST: Cognitive Remediation and Emotion Skills Training
DERS: Difficulties with emotion regulation scale
DSM: Diagnostic and Statistical Manual of Mental Disorders
ED: Eating disorder
EDE-Q: Eating Disorder Examination Questionnaire
ED NOS: Eating disorder not otherwise specified
EDs: Eating disorders
ERGT: Emotion regulation group therapy
ES: Effect size
MADRS-S: Montgomery Asberg depression rating scale – self-report version
TAS: Toronto Alexithymia scale
TAU: Treatment as usual
